# A Review of Recent Developments in Analytical Methods for Determination of Phosphorus from Environmental Samples

**DOI:** 10.3390/molecules30051001

**Published:** 2025-02-21

**Authors:** Tumelo M. Mogashane, Odwa Mapazi, Moshalagae A. Motlatle, Lebohang Mokoena, James Tshilongo

**Affiliations:** 1 Analytical Chemistry Division, Mintek, Private Bag X3015, Randburg 2125, South Africa; odwam@mintek.co.za (O.M.); moshalagaem@mintek.co.za (M.A.M.); lebohangm@mintek.co.za (L.M.); jamest@mintek.co.za (J.T.); 2 School of Chemistry, University of the Witwatersrand, Private Bag 3, Johannesburg 2050, South Africa

**Keywords:** phosphorus, eutrophication, spectrophotometry, environmental samples, colorimetric methods

## Abstract

Phosphorus is essential to environmental systems because it affects both agricultural productivity and ecological balance. Since it contributes to eutrophication and pollution problems, its existence in a variety of environmental matrices, including soil, water, and air, necessitates precise and effective determination methods for monitoring and managing its levels. This review paper provides an extensive overview of the latest advancements in analytical techniques for measuring phosphorus in environmental samples. We investigate sophisticated spectroscopic, chromatographic, and electrochemical techniques in addition to conventional approaches like colorimetric analysis. Innovative techniques such as mass spectrometry (MS), X-ray fluorescence (XRF) spectrometry, and nuclear magnetic resonance (NMR) spectroscopy are also highlighted in this study, along with newly developed technologies such as biosensors, lab-on-a-chip devices, and nanotechnology-based techniques. Real-time and field-deployable monitoring technologies are also covered, with a focus on their advantages and usefulness. Among the techniques reviewed, XRF and colorimetry methods have proven to be the most reliable due to their precision, cost-effectiveness, and adaptability for different sample matrices. While emerging spectroscopic and electrochemical techniques offer promising alternatives, further validation and standardization are needed for routine environmental monitoring. Future research should focus on integrating automated and high-throughput techniques to enhance monitoring capabilities further.

## 1. Introduction

Phosphorus is an essential nutrient for both plant and animal life and is involved in several biological processes [1,2]. On the other hand, an abundance of phosphorus in the environment, mainly from wastewater effluents, industrial discharges, and agricultural runoff can cause serious ecological problems, including eutrophication in aquatic systems [2]. Although phosphorus is a necessary ingredient for biological systems, excessive phosphorus accumulation in environmental matrices, such as soil, water, and air, causes serious ecological risks [3]. Excess phosphorus causes eutrophication in aquatic systems, which results in algal blooms that reduce oxygen levels and disturb aquatic life. While phosphorus-containing aerosols contribute to particulate pollution in the atmosphere, phosphorus accumulation in soil can change microbial activity and decrease nutritional availability [4]. Although lethal levels vary by ecosystem, excessive fertilization in soil can cause runoff contamination, and concentrations above 0.02 mg/L can cause eutrophication in water [5]. Its extensive distribution throughout ecosystems is facilitated by transport mechanisms like leaching, erosion, and volatilization, and its primary sources include atmospheric deposition, industrial effluents, wastewater discharge, and agricultural runoff [6]. Phosphorus levels in environmental samples must be accurately determined in order to be monitored and managed to avoid negative effects on ecosystem health and water quality [7,8,9].

Phosphorus is an essential part of phospholipids, Ribonucleic acid (RNA), Deoxyribonucleic acid (DNA), and Adenosine triphosphate (ATP), which are necessary for cellular membrane construction, energy transmission, and the storage of genetic information [10]. It is an essential nutrient in agriculture that supports plant growth and development, which is why it is a basic ingredient in fertilizers. It is equally important in natural ecosystems, where it maintains the productivity of terrestrial and aquatic food webs [8,11]. Furthermore, as phosphorus has a major impact on ecosystem production and soil fertility, its precise measurement is essential for academic study as well as real-world applications in environmental management and agriculture [12].

The demand for more sensitive, precise, and quick approaches has led to significant improvements in phosphorus determination analytical methods in recent years [11,13,14]. These advances cover a variety of techniques, such as chromatographic, electrochemical, spectrophotometric, and colorimetric procedures [15,16,17]. With an emphasis on the enhancements in detection limits, precision, and application to diverse environmental matrices, this review seeks to give a broad overview of these recent developments. Researchers and legislators can effectively handle the problems related to phosphorus pollution and its environmental management by being aware of the most recent developments and trends in the field of phosphorus analysis [4,17,18]. A thorough evaluation of these recent advancements is given in this review paper, which also discusses the difficulties and new directions in the field of phosphorus analysis. Improvements in sensitivity, accuracy, and detection limits are highlighted. This review paper provides insights into the most efficient, dependable methods for environmental monitoring and supports better informed environmental management and policy decisions by looking at the most recent approaches. By evaluating the accuracy, sensitivity, and applicability of these analytical methods for phosphorus determination, the review provides valuable insights for environmental authorities in improving monitoring frameworks and regulatory compliance. Additionally, it supports research institutions in developing innovative methodologies for phosphorus assessment, ultimately contributing to sustainable environmental management and policy development.

**Figure 1:**
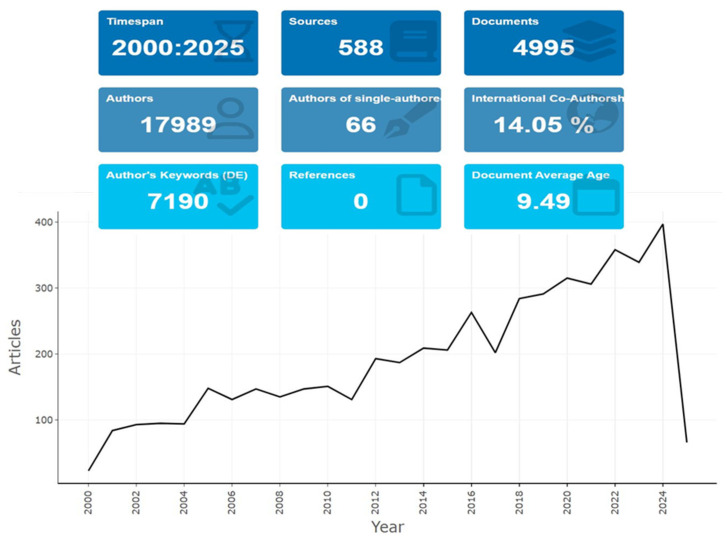
Number of articles according to PubMed database.

This review examines recent advancements in analytical techniques for phosphorus determination from environmental samples, drawing from peer-reviewed journal articles published between 2000 and 2025 (Figure 1). The literature was gathered from PubMed databases, focusing on the accuracy, sensitivity, and applicability of various techniques. The review follows a structured approach, beginning with an overview of phosphorus sources and environmental impacts, followed by a detailed discussion of traditional and emerging analytical methods, including spectroscopic and chromatographic techniques. It further explores quality assurance and control measures, data interpretation strategies, and the latest field-deployable methods. The review concludes with a comparative analysis of techniques and recommendations for future research and regulatory improvements.

## 2. Sources and Forms of Phosphorus

In environmental samples, phosphorus comes from man-made and natural sources. It exists in a variety of chemical forms that affect how it behaves and affects ecosystems [1,19]. Phosphorus is found naturally in soils and water bodies from the weathering of phosphate-bearing rocks and the breakdown of organic waste. However, the amount of phosphorus in the environment has increased drastically because of human activities [14,20,21]. Animal dung and fertilizers found in agricultural runoff are a significant cause of phosphorus pollution. Furthermore, the outflow of wastewater from residential and industrial sectors raises phosphorus levels in water streams [4]. Phosphorus is introduced by these inputs in both dissolved and particulate forms, with varying bioavailability and environmental effect potentials [22,23].

Phosphorus is commonly present in environmental samples in two primary forms: inorganic and organic. The most bioavailable form of phosphorus is inorganic, also known as orthophosphate, which is absorbed directly by microbes and plants [11,23]. It contains a variety of species, depending on the pH of the surrounding environment, such as phosphate ions (PO_4_^3−^, HPO_4_^2−^, and H_2_PO_4_^−^). However, microbial biomass remains of plants, and live or dead organisms are sources of organic phosphorus [1,10,24]. For biological absorption to occur, this form must be mineralized by microbial action [1]. Factors including soil type, land use, hydrology, and the presence of other nutrients affect the transit and destiny of phosphorus in the environment [13,25]. Excess phosphorus in aquatic systems can cause eutrophication, which can destroy aquatic life and result in toxic algal blooms and oxygen deprivation. To control phosphorus levels and to lessen its negative effects on ecosystems and water quality, it is essential to comprehend the sources and types of phosphorus in environmental samples [14,26,27].

## 3. Environmental Impact and Significance of Phosphorus

**Figure 2:**
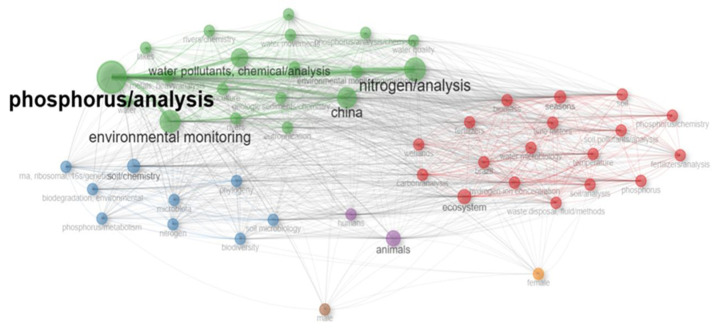
Clustered co-occurrence map to highlight key impacts and trends in phosphorus contamination.

Phosphorus contamination significantly impacts water, soil, air, and human health, necessitating a comprehensive assessment [28]. In soils, phosphorus accumulation alters nutrient cycling, reduces microbial diversity, and contributes to leaching, further contaminating water bodies. Atmospheric phosphorus, often released through agricultural dust, combustion, and industrial emissions, contributes to particulate matter pollution, affecting air quality and nutrient deposition in distant ecosystems [3]. Kidney dysfunction, cardiovascular illnesses, and metabolic problems are associated with human exposure to elevated phosphorus levels, mostly through contaminated air and water. It is essential to conduct further research on these effects to improve environmental laws and create mitigation plans [22]. A clustered co-occurrence map highlighting significant effects and patterns in phosphorus contamination is presented in Figure 2.

Furthermore, phosphorus has a significant effect on the environment, especially in aquatic habitats. Overabundance of phosphorus inputs can cause eutrophication, which is the excess of nutrients in water bodies that causes an increase in algal blooms [10,27,29]. Fish and other aquatic species may die because of hypoxia brought on by these blooms, which can lower the oxygen content of the water [13,14,30]. Algal biomass breakdown emits chemicals that can be dangerous to humans and aquatic life, aggravating oxygen loss even further [31,32]. Phosphorus pollution is not limited to eutrophication; it can also upset the biological balance, change the makeup of species, and deteriorate water quality [23,33]. Thus, regulating the amount of phosphorus in the environment is essential to preserving the health of ecosystems, guaranteeing the safety of drinking water, and promoting sustainable farming methods [32,34]. Although phosphorus is necessary for life, it must be managed in the ecosystem to avoid negative ecological effects [6,16,35]. To safeguard and maintain our natural resources, it is crucial to have a thorough understanding of the origins, forms, and environmental importance of phosphorus [25]. This includes creating monitoring programs and remediation plans [24,31,32,36].

A study by Amann et al. [32] examined the environmental effects of recovering phosphorus from municipal wastewater. Using the life cycle analysis technique, the study assessed 18 phosphorus recovery technologies for their potential to cause acidification, global warming, and cumulative energy demand [32]. The overall environmental performance of these indicators was then calculated by comparing them with other environmental parameters, such as recovery potential, organic and heavy metal micro-pollutant decontamination potential, and fertilizer efficiency. According to the Life Cycle Assessment (LCA), implementing phosphorus recovery from wastewater might result in a wide range of changes in gaseous emissions and energy demand [32]. Table 1 provides a clearer and more structured review of phosphorus impacts across different environmental matrices and emphasizes the significance of phosphorus in different ecological and economic situations as well as its impact on the environment.

## 4. Sample Collection and Preservation

A systematic process is required to ensure representativeness and accuracy while collecting soil and sediments samples for phosphorus assessment [41]. Usually, a soil auger or coring equipment is used to collect soil samples [42]. A range of instruments, including grabs, dredges, and sediment corers, can be used to gather sediment samples from rivers, lakes, or marine habitats [42]. Grab samples of surface sediments can be obtained with tools such as an Ekman grab or a Van Veen grab [41]. By using these techniques, soil or geological samples are gathered with the assurance that they are trustworthy and representative for further scientific examination and interpretation [18]. Surface water bodies, groundwater, or wastewater sources can all provide water samples for phosphorus analysis. Samples for groundwater are usually taken with a bailer or a submersible pump [30,43]. While less frequent, air samples can also be obtained for the study of phosphorus, especially in research on atmospheric deposition. High-volume air samplers fitted with filters (such as Teflon or glass fiber filters) that capture particulate phosphorus can be used to gather particulate matter in the air. Passive samplers can also be used to gather phosphorous over extended periods of time [15].

Various international standards, such as International Organization for Standardization (ISO) 5667 (Water Quality—Sampling), EPA 365.1 (Phosphorus Determination), and standard methods for the examination of water and wastewater (APHA, AWWA, WEF), provide guidelines for sample collection and preservation in different water matrices [40]. To prevent contamination and phosphorus species deterioration, these guidelines outline appropriate sample procedures, container materials, preservation measures (such as acidity and refrigeration), and holding periods [44]. To improve the consistency and dependability of analytical results across investigations, this section should be expanded to cover particular regulatory requirements for wastewater, acid mine drainage, surface water, and groundwater [1].

To avoid phosphorus loss or transformation, which could affect the analysis’s accuracy, samples must be properly preserved [41,45]. Samples should be kept cool (e.g., in a cooler with ice packs) and sent to the laboratory as soon as feasible if immediate drying is not possible [41]. To avoid changes caused by moisture, long-term storage should be conducted at room temperature in a dry atmosphere [41]. To prevent microbial activity and chemical reactions that could change the phosphorus level, unfiltered samples are frequently acidified with sulphuric acid to a pH of less than 2 for total phosphorus analysis [7]. Table 2 provides a clear reference for sample collection and preservation across different water matrices, ensuring accurate phosphorus determination.

## 5. Extraction Techniques

The amount of phosphorus present in soils, sediments, and other matrices is measured using a variety of extraction procedures [8]. These approaches can be divided into three categories: sophisticated extraction methods, physical methods, and chemical extraction. Chemical extraction techniques are extensively employed because of their ease and specificity [8]. Sodium bicarbonate is used in the Olsen method, which yields accurate measurements of accessible phosphorus and works well for alkaline soils [51]. Ammonium fluoride and hydrochloric acid are used in the Bray and Kurtz P1 method, which effectively removes phosphorus that is attached to soil particles and is appropriate for acidic soils [25]. The highly adaptable Mehlich-3 extraction method is a universal technique that extracts phosphorus from a variety of soil types using a combination of acids and chelating agents [30,52]. Morgan extraction is another noteworthy technique that targets phosphorus in neutral to slightly acidic soils by using sodium acetate buffered at pH 4.8. These chemical procedures use chemicals to solubilize phosphorus so that it can be quantified later using spectroscopic or colorimetric methods [53].

Sequential extraction, which uses variety of reagents, separates phosphorus into several forms, including soluble, exchangeable, and organic phosphorus [54]. This technique yields comprehensive data on the speciation and distribution of phosphorus inside a sample [6,55]. A common remediation method for contaminated soils is soil washing, which is agitating the soil with water or chemical solutions to remove excess phosphorus [52]. By using ultrasonic waves to break down soil aggregates, release bound phosphorus, and improve extraction yield, ultra-sonication improves the efficiency of chemical extractions.

A study by Yang et al. [51] used an improved standard addition method to measure the phosphorus (P) content of soil using Inductively Coupled Plasma Optical Emission Spectroscopy (ICP-OES). Based on the conventional standard addition method (CSAM) and calibration curve method (CCM), ICP-OES developed an improved standard addition method (ISAM) for the determination of phosphorus in soil in their study [51]. Each method’s detection limit, quantification limit, and recovery rate were computed; the soil sample and blank recovery rates fell between 90% and 110% of the range. The results from ISAM and CCM did not significantly differ from one another according to the t-test (*p* > 0.05), and the relative errors between the two sets of data were all within 10%. As a result, the suggested method somewhat mitigated the matrix effect and was deemed suitable for quick and precise batch analysis of P content in soil samples, particularly those with a clear matrix effect [51].

More accurate, eco-friendly, and effective extraction processes have been made possible by advancements in extraction techniques [6,56]. By heating the extraction solvent and sample with microwave energy, microwave-assisted extraction (MAE) improves efficiency and drastically cuts down on extraction times and solvent use [53]. Supercritical fluid extraction (SFE) is an environmentally friendly substitute for conventional procedures in the extraction of organic phosphorus compounds [57,58]. It works by using supercritical carbon dioxide (CO_2_) that has been modified with co-solvents. High pressure and temperature are used in accelerated solvent extraction (ASE) to improve extraction efficiency and cut down on extraction time and solvent use [8]. Electrochemical techniques, which are frequently combined with chemical techniques to improve recovery, use electric fields to mobilize and remove phosphorus [10]. High specificity, automation, and miniaturization are all possible using nanotechnology-based phosphorus extraction and binding techniques that use nanoparticles [18]. Improvements in extraction technologies can lead to notable gains in terms of efficiency, environmental impact, and analytical performance. Table 3 presents a succinct and straightforward comparison of different phosphorus extraction methods, emphasizing their salient features, benefits, and drawbacks for investigation of environmental samples.

### 5.1. Colorimetric Methods

Because colorimetric methods are both easy to use and affordable, they are essential for phosphorus analysis in environmental research, agriculture, and water quality monitoring [2,69]. Conventional colorimetric methods, such the molybdenum blue method, are frequently employed because of their dependability and simplicity of use [38,70]. In this process, an acidic medium forms a phosphomolybdate complex, which is then reduced with a reducing agent such as ascorbic acid to produce a blue-colored complex [70]. A spectrophotometer is used to quantify the blue color intensity, which corresponds to the phosphorus concentration in the sample [2,71]. The method is highly recognized for its accuracy and sensitivity in quantifying orthophosphate, which is the phosphorus form that is most bioavailable [72].

The Molybdenum Blue Method is one of the most used methods [9,70,73]. Using this procedure, the sample is treated with an aqueous solution of ammonium molybdate, which combines with the phosphorus to generate a phosphomolybdate complex in an acidic environment [56,70]. After that, a reducing agent like ascorbic acid reduces this complex, producing a blue-colored molecule. Using a spectrophotometer, the blue color’s intensity may be directly related to the amount of phosphorus present [9,47,70]. After proper digestion, this very sensitive approach can be used to analyze both organic and inorganic phosphorus [73,74].

Phosphorus reacts with vanadate and molybdate ions in an acidic solution to generate a yellow-colored compound using the vanadomolybdo-phosphoric acid technique [2,70,73]. Although this approach is quicker and easier to use than the molybdenum blue method, it is often less sensitive. In cases when elevated phosphorus concentrations are anticipated, it is frequently applied to soil and plant tissue samples. Ammonium molybdate is also used in the Ascorbic acid method to generate a blue-colored complex [70]. The distinction can be attributed to the reducing agent employed, ascorbic acid, which is preferred due to its ease of use and consistency in a variety of environmental media, such as soil and water extracts [73].

A study by Wieczorek et al. [2] examined the phosphorus-containing substances found in plant tissues using modern instruments, analytical chemistry, and calorimetry. They employed both classical absorption flame atomic absorption spectroscopy (FAAS) and Inductively Coupled Plasma Atomic Emission Spectroscopy (ICP-AES) techniques as well as sophisticated spectrophotometric studies [2]. The use of combination techniques, such as electro-thermal vaporization inductively coupled plasma atomic emission spectrometry (ETV-ICP-AES), has significantly advanced these types of analytical efforts by enabling the direct detection and isolation of phosphorus analytes in plant tissues in a “one step” analytical process. NMR, particularly the ^31^P NMR technique, is now considered the most universal analytical tool allowing the determination of various chemical forms of plant phosphorus both qualitatively and quantitatively, presumably due to its higher limit of detection [2].

#### 5.1.1. Innovations and Improvements in Colorimetric Analysis

Sensitivity, accuracy, and usability of colorimetric analysis for phosphorus in environmental samples have been greatly improved by recent breakthroughs and advancements [9,71,75]. Reagent chemistry advancements have produced less hazardous and more stable reagents, which have improved safety and the environment [76]. The development of automated and microfluidic devices has reduced human error and enabled high-throughput analysis. Modern spectrophotometric detectors, which offer more accurate readings at lower detection limits, are frequently incorporated into these systems [2,77]. Additionally, smartphone-based colorimetric measurement and digital image processing have come together to allow for minimum equipment and instantaneous findings during in-field testing [37]. These developments in technology, along with enhancements in instruments and reagent composition, have improved colorimetric analysis’s efficiency, dependability, and accessibility for tracking phosphorus in a variety of environmental matrices [36]. Furthermore, to reduce interferences from other compounds in complicated matrices, new reagents and techniques have been developed, improving the robustness and versatility of colorimetric phosphorus determination [77]. Additionally, colorimetric kits that are field-deployable and portable have been created, allowing for on-site analysis without the need for complex laboratory equipment. While colorimetry has been used for decades, recent innovations have significantly improved sensitivity, accuracy, and field applicability. To improve detection limits and reduce interference from complicated matrices, modern kits now include improved reagents, smartphone integration, and microfluidic technologies. Additionally, portable spectrophotometers and digital analysis tools have improved result consistency and eliminated subjective interpretation. These advancements make current kits more robust, user-friendly, and adaptable for real-time environmental monitoring compared to their predecessors [75].

#### 5.1.2. Applications and Limitations of Colorimetric Methods

Colorimetric methods are widely used for phosphorus analysis in environmental monitoring of water bodies, soil fertility evaluations, and wastewater treatment procedures [38]. These techniques are very helpful in agricultural research since they maximize fertilizer use and stop phosphorus runoff from causing eutrophication in aquatic ecosystems. Colorimetric approaches offer advantages and are widely used, but they also have drawbacks [2,38]. Their vulnerability to interference from other materials in the sample, such as silicates, arsenates, and organic debris, is a major drawback that may affect the accuracy of the findings [77]. Furthermore, the overall phosphorus level, which includes organic and particle forms, may not be accurately represented by current methods, which are normally restricted to assessing inorganic orthophosphate [37]. Even if established colorimetric methods, such as the molybdenum blue method, are still fundamental to the measurement of phosphorus, continuous advancements in technology are improving the methods’ performance and usefulness [75]. The colorimetric method remains a highly viable option for phosphorus determination due to its simplicity, cost-effectiveness, and adaptability for both laboratory and field applications. It is suitable for routine environmental monitoring, particularly in environments with limited resources, because it requires less equipment than sophisticated spectrometric approaches. The precision and reproducibility of the procedure have increased due to recent developments in automation, detection limits, and reagent stability [78]. Furthermore, colorimetric assays can be modified for different species of phosphorus, offering a thorough understanding of the dynamics of phosphorus in the environment. The resilience of the approach makes it possible to analyze big sample sets quickly, which is crucial for pollution management and regulatory compliance [77]. Furthermore, it is now more applicable for in situ and real-time measurements due to its integration with microfluidic devices and portable digital readers. Colorimetric methods continue to be a mainstay in phosphorus analysis despite several drawbacks, offering crucial information for water quality evaluation, agricultural management, and environmental protection [38].

### 5.2. Digestion Methods

It is necessary to transform organic phosphorus molecules into inorganic forms for total phosphorus analysis [54,74]. Potassium persulfate is frequently employed as an oxidizing agent in persulfate digestion, a process that involves heat and pressure [19]. All types of phosphorus are transformed into orthophosphate by this mechanism, which allows for colorimetric measurement [54]. Water, soil, and sediment are just a few of the environmental samples that can benefit from persulfate digestion [53]. Samples of soil and silt are dissolved in aqua regia, a solution made of strong hydrochloric acid and nitric acid, which releases bound phosphorus [8]. The phosphorus content is then determined using colorimetric or other analytical techniques, which are useful for decomposing complicated matrices [57,76].

A study by Sichler et al. [10] compared several aqua regia digestion techniques with ICP-OES, Inductively Coupled Plasma Mass Spectrometry (ICP-MS), and photometric determination to ascertain the phosphorus level in sewage sludge [10]. All possible method (digestion and measurement) combinations were used to examine 15 sewage sludges from 11 different wastewater treatment facilities. Additionally, a single sewage sludge was investigated in a 28-person inter laboratory comparison (ILC) [10]. The results of their study indicated that there are some significant differences between the aforementioned approaches; however, after normalization to the grand mean (the average of 15 sludge’s), phosphorus recovery was shown to range between 80 and 121% for all method combinations and sludge’s [10]. The results of the ILC and the analysis of 15 sludge’s were essentially identical. ICP-OES measurements reveal larger phosphorus contents than ICP-MS and photometric phosphorus determination, and there is a trend for higher phosphorus recovery during microwave digestion as opposed to reflux digestion. The study recommends ICP-OES measurement of phosphorus in sewage sludge following microwave digestion [10]. The components of digestion and colorimetric methods for phosphorus analysis in environmental samples are listed in Table 4.

### 5.3. Sequential Extraction Methods

Phosphorus is fractionated into several chemical forms using sequential extraction techniques, which sheds light on the element’s environmental mobility and bioavailability [37,79]. A sequence of extractions using reagents of increasing strength are required for this technique [63]. Different phosphorus fractions, including those that are loosely bound, iron-bound, aluminum-bound, calcium-bound, and organic phosphorus, are the focus of each phase [10,80]. After extraction, the fractions are subjected to colorimetric analysis or other methods of analysis [54]. These wet chemistry techniques provide accurate and sensitive ways to detect the amounts of phosphorus in a variety of matrices [63,80]. Every technique has unique uses, benefits, and drawbacks that enable customized selection according to the kind of sample and the goals of the study. Table 5 provides a useful comparison of phosphorus concentrations in environmental samples from various global regions. It includes information on the extraction time, percentage recovery, extraction method, and measuring method.

## 6. Spectroscopic Methods

**Figure 3:**
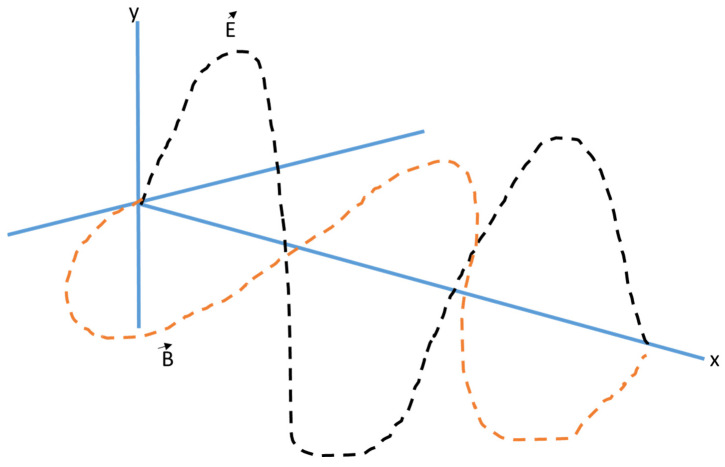
Electromagnetic radiation wave with a change in the magnetic field.

Spectroscopic methods are used to quantify how electromagnetic radiation interacts with materials [83,84]. Because each spectroscopic technique has unique benefits, restrictions, and uses, they are useful instruments for both industry and scientific study. Chemical researchers have an interest in characterizing phosphorus in materials such as phosphates and phosphides [83]. Qualitative spectroscopy studies the radiation absorbed or emitted by an atom or molecule, which will give information about the composition of phosphorus [32,85]. Quantitative spectroscopy gives information on the number of absorbing or emitting atoms or molecules by measuring the total amount of radiation [84,86]. Electromagnetic radiation is a type of energy that is made up of an electric and magnetic field, as depicted in Figure 3 below. It consists of a perpendicular magnetic vector and an electric vector that oscillate in the same plane perpendicular to the direction of propagation. The primary range of the electromagnetic spectrum spans from shorter wavelengths to longer wavelengths, such gamma rays [85].

Different types of spectroscopic techniques include absorption, emission, and scattering. The range in which a material absorbs electromagnetic radiation is used in absorption spectroscopy [84,85]. It involves many chemical techniques like infrared (IR) spectroscopy and ultraviolet (UV) spectroscopy, as well as atomic absorption spectroscopy [84]. This technique is based on the Beer-Lambert law, which states that the sample concentration and path length for a given substance are directly correlated with the light absorption. The scattering process is far faster than the absorption/emission process. Among the most useful uses of light scattering spectroscopy is Raman spectroscopy [87]. These spectroscopic methods provide trustworthy tools for the accurate and exact measurement of phosphorus in a range of sample matrices [36].

### 6.1. Ultraviolet-Visible Spectroscopy

**Figure 4:**
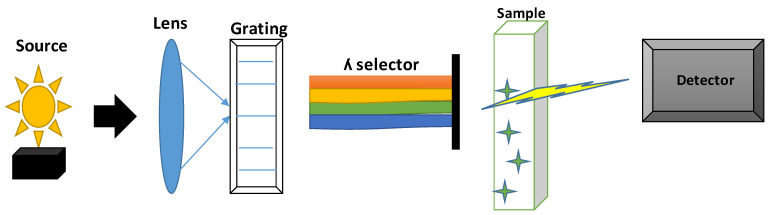
UV-Vis spectroscopy instrumentation setup.

Ultraviolet-Visible (UV-Vis) spectroscopy is an analytical technique used to measure the absorption or reflectance of light in the ultraviolet and visible regions of the electromagnetic spectrum [88]. Electronic changes from one energy level to another are caused by molecules absorption of light at particular wavelengths [89]. The quantity of light that enters the sample unabsorbed is known as its transmittance [90]. Light Source provides both visible and ultraviolet light; usually, tungsten is used for visible light and deuterium for ultraviolet light. A monochromator permits the selection of a particular wavelength to pass through the sample by dispersing light into its constituent wavelengths. The detector determines the light’s intensity, either reflected or transmitted [44,89]. Figure 4 shows UV-Vis spectroscopy instrumentation setup.

A study by Zamparas et al. [91] investigated the iron (Fe) ions, which were incorporated into the bentonite’s interlayer space to create a modified inorganic bentonite (Zenith/Fe), also known as Zenith-N, which was derived from natural bentonite [91]. The effectiveness of this material as an adsorbent for phosphate removal from natural waters was investigated using bench-scale batch studies. Adsorption kinetics and adsorption isotherms were used to assess phosphate uptake in relation to pH. Additionally, the impact of temperature and salinity (fresh, brackish, and marine waters) on the ability to absorb phosphate was studied [91]. Experiments were conducted to compare the performance of the commercial product Phoslock (lanthanum-modified clay) with unmodified bentonite, known as Zenith-N. The findings demonstrated that Zenith/Fe’s phosphate-uptake capacity (11.15 mg/g, at pH = 7) was equivalent to that of unmodified bentonite 11.60 mg/g of phospholock. Within an hour, approximately 80% of the phosphate in the water was eliminated, according to adsorption kinetics [91].

In the fertilizer industry and in agriculture, phosphate analysis is essential, especially for the manufacture of fertilizer and the health of the soil [92]. A study by Vieira et al. [93] determined the phosphorus in fertilizers using laser-induced breakdown spectroscopy (LIBS) spectroscopy, (SD-LIBS) by hyphenation with a spark discharge method to increase the sensitivity of P measurement in fertilizers [93]. Preliminary experiment results indicated that the P (I) line at 214.9 nm, which was unaffected by Fe, Cu, and Zn, was the best wavelength to measure P [93]. A homemade high-voltage electronic circuit with two cylindrical tungsten electrodes at an ideal output voltage of 4.5 kV and tips positioned at the ideal separations of 4 mm between them and 2 mm above each other produced the electrical discharge [16]. For the purposes of environmental protection, regulatory compliance, and quality control, phosphate analysis is crucial in a variety of sectors [65,92].

A study by Vinod et al. [17] assessed FTIR spectroscopy in a new multi-model assessment of phosphorus in coal and its ash. Phosphorus in coal and coal ash can only be detected using precise and sensitive technology, which is necessary for effective monitoring [17]. Although coal and coal ash samples have very mild fluorescence intensities, XRF is a frequently used analytical technique for determining their phosphorus content. In their study, they investigated the possibilities of using machine learning models (support vector regression (SVR), random forest (RF), piecewise linear regression (PLR), and partial least square regression (PLSR)) in conjunction with FTIR spectroscopy to measure the phosphorus content of coal and coal ash [17]. To quickly and accurately estimate the low levels of phosphorus in coal and its ash, their research suggests a multi-model estimation approach that uses FTIR spectral data and PLR, PLSR, and RF. According to their findings, FTIR in conjunction with a multi-model approach that combines RF, PLR, and PLSR regression models is a trustworthy tool for measuring phosphorus in coal and coal ash quickly and accurately [17]. Depending on the industry, sample type, and the required sensitivity, many analytical procedures use UV-Vis for phosphorus analysis [94]. The advantages of using UV-Vis technique is that it is cost-effective when considering alternative analytical techniques such as ICP-OES, ICP-MS, or UV-Vis spectrophotometry [65].

### 6.2. Atomic Absorption Spectroscopy (AAS)

AAS is used to quantify a specific metal element in a sample. It measures the concentration of gas-phase atoms by using light absorption. The basis of AAS is the idea that light at a certain wavelength can be absorbed by free atoms in their ground state [95]. The concentration of the element in the sample directly correlates with the amount of light absorbed. The necessary wavelength is emitted by a hollow cathode lamp designed specifically for the element being measured [96]. The atomizer utilizes a flame or graphite furnace to break the sample up into free atoms, while the monochromator separates the particular light wavelength that the target atoms absorb [95,97]. Lastly, the detector determines the light’s intensity both before and after it enters the sample. The variation in intensity reveals the degree of absorption and, consequently, the element’s concentration.

Phosphorus is becoming more and more interesting, and thus many analysts are turning to atomic absorption attributed to the minimal interruptions. Phosphorus is less sensitive than other elements that are typically evaluated by flame AAS [98]. Under vacuum ultraviolet light, the most sensitive phosphorous resonance lines can be found. There are two methods available for digesting materials; dry ashing comes first, then dilution by acid solubilization [99]. At 1100 °C or above, some compounds containing phosphate and phosphide boil or separate [98]. The vacuum ultraviolet area is where the most sensitive phosphorous resonance lines are located [94]. Improved repeatability can be achieved by short integration periods, aspirating distilled water between samples and standard solutions, and increasing oxidant flow above the minimum [100].

Phosphorus has a typical concentration and detection limit of 125 µg/mL and 40 µg/mL, respectively, when measured with an electrical double layer (EDL) [101,102]. It is advisable to utilize other methods of analysis for values below 400 µg/mL to reduce phosphorus volatilization. The vacuum ultraviolet area is where the most sensitive phosphorous resonance lines are located [102]. It is therefore necessary to utilize a less sensitive non-resonance line. For flame analysis, the typical range of phosphorus concentrations is 400–8000 µg/mL. A study by Sai et al. [102], investigated the use of a one-step solvothermal approach to report rod-like phosphorus-containing metal complex aggregates (CePn) [102]. The results demonstrate that the addition of 4 weight percent of CePn reduces the peak heat release rate (PHRR) and total smoke release (TSR) of the bulk PC by 46% and 26%, respectively, due to CePn’s dual-phase flame retardant mechanisms [102].

### 6.3. Inductively Coupled Plasma Optical Emission Spectroscopy

**Figure 5:**
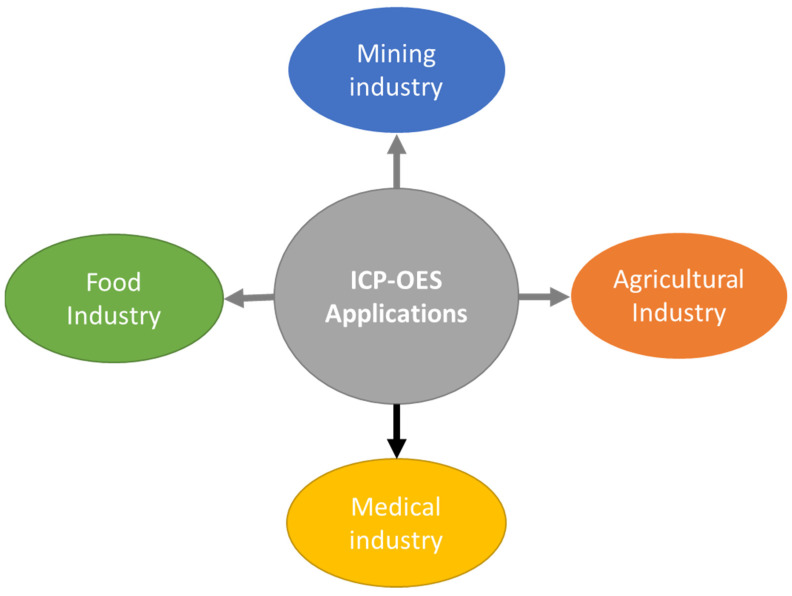
The analytical application of ICP-OES.

ICP-OES is an analytical method for determining the element concentrations in a variety of samples, such as liquids, solids, and gases [103,104]. An inductively linked radiofrequency (RF) field creates a high-temperature plasma, usually around 10,000 K, into which the sample is inserted. An electrically conductive gas called a plasma is made up of ions, electrons, and neutral particles. The sample is atomized, excited, and desolvated (if in solution) upon entry into the plasma [103]. The electrons in the atoms of the elements present in the sample are excited by the high energy of the plasma. Upon returning to their ground state, excited electrons release light at certain wavelengths that are unique to each element. The light that is released has an intensity that is directly related to the element’s concentration in the sample [105,106]. Detection and quantification is carried out by the optical system, wherein the spectrometer receives and directs the light that the elements emit [106]. The spectrometer uses a prism or diffraction grating to split the light into its individual wavelengths. A charge-coupled device (CCD) or photomultiplier tube (PMT), which gauges light strength at each wavelength, is used to detect the scattered light. Standards with known amounts of the relevant components are used to calibrate the device. Plotting the light’s intensity versus its concentration yields a calibration curve [106]. By comparing the emission intensities of unknown materials to the calibration curve, the concentration of each element can be ascertained. Applications include environmental analysis, which involves monitoring environmental conditions for pollution and regulatory compliance by tracking trace constituents in samples of air, water, and soil [107]. The pharmaceuticals sector monitors elemental impurities in raw materials, intermediates, and final products [104,108]. The metals and mining industry explores the complexities of metals, alloys, and ores, as shown in Figure 5 below.

ICP-OES is a widely used method for determining the amount of phosphorus present in a variety of samples, such as biological, environmental, and industrial materials [9]. There is also a thorough discussion of topics such as phosphorus speciation, the molybdenum blue method, organic phosphorus species digestion processes, selecting model compounds for analytical research, quality control, and the accessibility of environmental certified reference materials (CRMs) for phosphate [65]. To guarantee reproducible results, the detection limits depend on the equipment and sample matrix; the low ppm range is usually where phosphorus is detected in ICP-OES [109]. However, this can vary due to its sensitivity, accuracy, and versatility in handling different kinds of samples [3].

### 6.4. Inductively Coupled Plasma Mass Spectrometry

**Figure 6:**
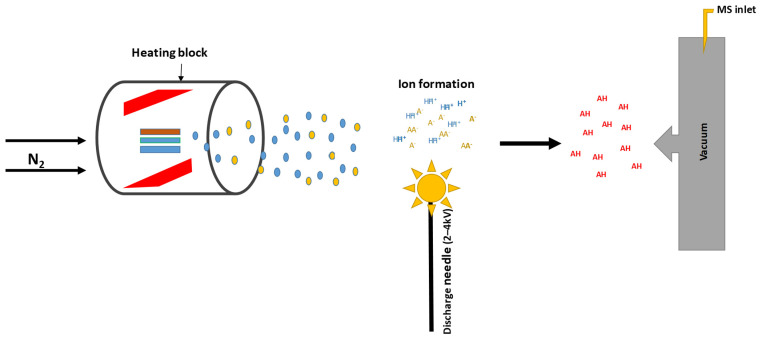
Schematic diagram of the ionization process in the ICP-MS.

ICP-MS is a sophisticated analytical method for identifying and measuring trace elements in a variety of samples [110,111,112]. It combines the advantages of mass spectrometry for ion detection and measurement with inductively coupled plasma for ionizing the sample. Techniques for vaporization or digestion are needed for solid materials [110]. An argon gas stream propels the aerosolized sample into a high-temperature plasma, which is between 6000 and 10,000 K. Then an inductively linked RF field produces the plasma [111]. The elements in the sample are atomized and ionized in the plasma, resulting in ions that are positively charged. Subsequent to being separated from the plasma, the ions are placed into a mass spectrometer. At this stage, they are separated by going through a magnetic field or a set of quadrupoles in accordance with their mass-to-charge ratio (*m*/*z*). A discrete dynode electron multiplier is used to identify the separated ions [110,112]. Figure 6 shows a schematic diagram of the ionization process in the ICP-MS.

Applications for ICP-MS include environmental analysis, which measures pollution and adherence to environmental regulations by tracking trace metals and other elements in water, soil, air, and biota [111,112]. ICP-MS is used for the analysis of trace elements in rocks, ores, and minerals to ascertain their composition and support mineral exploration [112,113]. ICP-MS is also used to determine isotopic ratios for materials such as plutonium and uranium, which is crucial for environmental monitoring and nuclear forensics [114]. One of the benefits of ICP-MS is the ability to identify components at incredibly low concentrations, frequently in the parts-per-trillion (ppt) or parts-per-billion (ppb) range. Moreover, it has the ability to quantify trace to large levels of element concentrations in a single sample. Its multi-element capability makes it effective for thorough elemental analysis by simultaneously detecting and quantifying numerous elements in a single analysis [112].

Peters et al. [115] used a screening method for the identification and description of nanoparticles in complex matrices, including food and biological tissues by single particle ICP-MS (spICP-MS). To calculate particle size, concentration, and size distribution from the raw data, a data evaluation tool was developed [115]. Both a sector-field equipment and a typical quadrupole instrument were used for spICP-MS measurements. The detection limits for the sector-field instrument size are lower, at 10 nm (Au). For 60 nm Au NPs, the concentration detection limits were 1 ng L^−1^, while for 500 nm SiO_2_, they were 0.1 μg L^−1^. These effects can be mitigated by methods such as matrix matching, internal standardization, or the application of a dilution factor [116]. By choosing other isotopes or mass ranges, as well as employing collision/reaction cell technology, these interferences can be reduced [117]. For phosphorus analysis, ICP-MS is a very sensitive and flexible method, especially where high precision and low detection limits are needed. Accurate phosphorus measurement is made possible by ICP-MS, which ionizes samples in a high-temperature plasma and analyses the resultant ions according to their mass-to-charge ratio. It is perfect for environmental investigations because of its capacity to handle complex matrices like water, soil, and sediments. However, difficulties like spectrum interferences and matrix effects, especially from polyatomic ions, call for rigorous method optimization and the application of suitable internal standards [112,117].

#### Mass Spectrometry Methods

Mass spectrometry techniques have become essential for determining the amount of phosphorus in environmental samples because of their remarkable sensitivity, specificity, and capacity to work with intricate matrices [118]. Phosphorus-containing organic compounds are identified and quantified using molecular MS techniques such as GC-MS and liquid chromatography-mass spectrometry (LC-MS), while total phosphorus can be precisely quantified using techniques like ICP-MS [119]. The ability of MS to separate, identify, and characterize phosphorus species in complex sample matrices is improved by linking it with chromatographic techniques, which offers comprehensive insights into their sources and transformations [6]. High-resolution MS and tandem mass spectrometry (MS/MS), two recent developments, considerably enhance detection limits and the capacity to examine low-abundance phosphorus species [118,119]. To overcome obstacles in environmental monitoring, like differentiating distinct phosphorus forms and comprehending their ecological effects, these integrated approaches are essential [119].

As early as 2002, ICP-MS was proven successful in the detection of phosphorus in the environment due to its sensitivity [48]. Compared to the traditional photometric method, ICP-MS had an advantage in that sample preparation was minimal. Real water samples only needed to be filtered to determine dissolved P, or analyzed directly after digestion to determine total P. Moreover, ICP-MS exhibited a much higher linear range (10–5000 ppb) compared to 8–150 ppb of the UV-vis photometric method. Because of this, notable differences were observed at higher sample concentrations, which were attributed to the dilutions that had to be made to suit the UV-vis method’s analytical range [48].

Catrina et al. [120] reported on a method for determination of P from different solid wastes ranging from incinerated medical waste, sewage sludge, vegetable waste, and sludge from meat processing [120]. Orthophosphate, polyphosphate, and organic phosphorus are the forms of P that are found in waste. Using CRMs, the method demonstrated the ability to analyze P in the different wastes, achieving LODs ranging from 21–33 ppb, LOQs 80–110 ppb, recoveries 95.2–98.9%, and relative standard deviation (RSD from intermediate precision) 1.43–2.10%. A microwave assisted acid digestion was performed for the dissolution of all waste types prior to ICP-MS analyses. Potential interferants were identified to be Fe, Cr, Cd, Pb, and Zn cations, and artificial P solutions containing different amount each of the interferants were prepared to evaluate the effect these have on P determination. At 250 ppb P and 450 ppb cation concentrations, P recoveries ranged between 91.2–96.0%, indicating the method’s selectivity [120]. In a first of its kind study, Fernandez et al. [121] demonstrated a method of removing these interfering species by using oxygen in the reaction cell to change the *m*/*z* ratio of P to 47 and then using another quadrupole mass analyzer to analyze phosphorus, giving an interference-free detection and improved detection limits [121]. This approach (tandem MS) overcomes interference by transforming ^31^P to ^31^P^16^O, which is different from interfering species such as ^15^N^16^O, ^14^N^16^O^1^H, and ^12^C^18^O^1^H and is measured on the second quadrupole mass analyzer [121]. Tandem MS has some advantages over the EPA recommended phosphomolybdenum blue method, including much lower detection limits, faster analysis time, and high throughput. This is especially true with the incorporation of the triple quadrupole technology into the ICP-MS [121]. Because of this, more and more researchers are exploring the technique for P detection in different types of samples [122,123].

Triple quadrupole (TQ) mass spectrometry is an example of tandem quadrupole mass spectrometry, one where three quadrupole mass analyzers are employed. Ions from the sample go into the first quadrupole (Q1). At this stage, they still represent the mass of the parent ion. From Q1 the ions go to Q2, also known as the collision cell, where they are subjected to radiofrequency (RF) voltage in the presence of an inert gas [123]. The applied RF voltage introduces oscillation that causes interaction between the ions and gases, which results in collision-induced dissociation, where the ions fragment into smaller ions of less molecular weight. These ions are known as daughter ions or product ions. They proceed to the last quadrupole (Q3) where a selection of product ions of a specific *m*/*z* takes place. The ion with the selected *m*/*z* is then filtered and gets to the detector to be recorded [122].

Yet with all the detection power that mass spectrometry has with its technologies such as TQ mass analyzers, researchers are still searching for more ways to get even more from it. This has been shown in several studies involving the detection of phosphorus-based herbicides in environmental samples by coupling the MS to a chromatographer [124]. For instance, Chen et al. [125] investigated the use of IC coupled to ICP-MS for the speciation of glyphosate, phosphate, and aminomethylphosphonic acid in soil extracts [125]. Their method was able to separate the analytes within 5 min in the column to feed into the ICP-MS, which returned LODs ranging from 1.0–1.5 µg L^−1^ (P). A similar study simultaneously detected the presence of glyphosate, glufosinate, fosamine, and ethephon in water using IC coupled to quadruple ICP-MS [125].

Similar to the work of Chen et al. [125], after complete separation of the herbicides, LODs ranging between 1.1–1.4 µg L^−1^ (compound) were achieved. These studies addressed the issue of carbon deposition on the ICP torch and cones when organic solvents were used in the mobile phase. However, the reported LODs may make the methods unsuitable for use in some parts of the world where the highest permissible glyphosate level is as low as 0.1 µg L^−1^ in drinking water [126]. Lajin and Goessler were able to achieve a much lower LOD (0.19 µg L^−1^, P) for glyphosate analysis. Their method utilized a TQ ICP-MS as a detector for a High-performance liquid chromatography (HPLC) to analyze glyphosate together with other herbicides such as aminomethylphosphonic acid (AMPA), glufosinate, fosamine, and ethephon in ground, tap, and river waters [126]. Generally, the LODs ranged from 0.14–0.27 µg L^−1^, which were a big improvement from the LODs reported from single quadrupole ICP-MS coupled to a chromatographer [124]. The reported LODs could be further improved by using oxygen gas in the collision/reaction cell (CRC). Lajin and Goessler [126] investigated the effect of different gases on the LODs calculated from the calibration curve using oxygen, helium, hydrogen, and no gas. Based on the calibration of 0.5–5.0 μg P L^−1^, the respective LODs obtained for the gases were 0.02, 0.36, 0.46, and 0.55 μg P L^−1^ [126].

## 7. Chromatographic Techniques

### 7.1. High-Performance Liquid Chromatography

HPLC is an advanced technique used for separating, identifying, and quantifying components in a mixture. It is widely employed in various fields, including pharmaceuticals, environmental analysis, food science, and chemical research [127,128]. The column is packed with a stationary phase (often-tiny particles with certain chemical characteristics); the column is the central component of the HPLC system [129]. The mobile phase, a liquid or occasionally a combination of liquids, passes through the column. This mobile phase is used to inject the sample, which is then passed through the column [130]. The components of the sample separate according to characteristics such as polarity, size, or charge because of their varied interactions with the stationary phase and the mobile phase [131]. A polar stationary phase and a non-polar mobile phase are used in normal-phase HPLC [129]. A polar mobile phase and a non-polar stationary phase are used in reverse-phase HPLC [129,132].

Phosphorus does not include chromophores or UV-absorbing groups, which are required for the typical HPLC detection procedures; phosphorus alone is difficult to detect by HPLC [119,133]. Nevertheless, if phosphorus-containing substances (such as phosphates, organophosphates, or phosphonates) can be derivatized or if they are a component of bigger molecules that absorb UV light, they can be examined using HPLC [119,134,135]. Prior to column derivatization to create observable species, phosphorus compounds can be chemically changed, or derivatized, prior to being injected into an HPLC system [136]. For instance, phosphorus can be changed into a derivative that absorbs UV light [119,137].

### 7.2. Ion Chromatography (IC)

**Figure 7:**
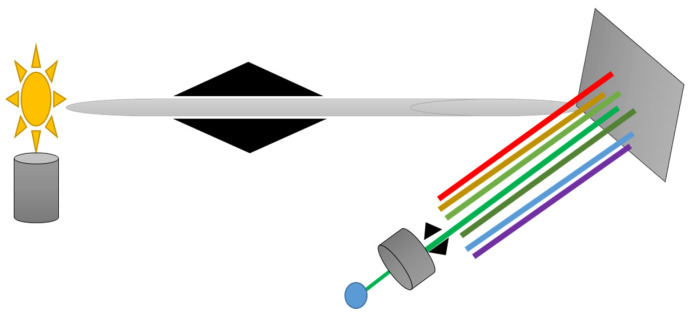
A schematic diagram for the UV detector used in IC.

Ion chromatography is mainly used for the separation and examination of ionic species, such as anions and cations. It is extensively utilized in food safety, water quality testing, environmental analysis, and pharmaceuticals [138]. The ion-exchange column, which features a stationary phase made up of charged resin particles, is the central part of an ion chromatography system [139,140]. Eluent, also known as the mobile phase, is usually a solution that speeds up the ion exchange procedure; this is typically sodium carbonate, potassium hydroxide, or sodium bicarbonate solutions [135]. The eluent’s composition and strength have an impact on the separation process. Depending on their charge and affinity for the resin, the sample ions exchange with the counter-ions on the resin as they go through the column and the ions separate and elute at different times [119,138]. The most used detection technique in ion chromatography is conductivity detection. It tracks the eluent’s conductivity as various ions move through the detector. Conductivity detection is the best method for detecting inorganic ions because it is sensitive to changes in ion concentration [138]. Figure 7 shows a schematic diagram for the UV detector used in IC.

Phosphorus can be effectively analyzed using IC, especially when the phosphorus is present as phosphate ions (PO_4_^3−^). Reliability and quality assurance for long-term sensing in complex contexts are still inadequate, despite the fact that several sensor technologies can reach nM detection levels [141]. Additionally, some sensors are sensitive to high concentrations of background ions. By highlighting current advancements in aquatic chemical sensors to track the amounts of nitrate (NO_3_^−^), nitrite (NO_2_^−^), ammonium (NH_4_^+^), and phosphate (PO_4_^3−^) ions in water [141]. PO_4_^3−^ is the most prevalent type of phosphorus that is examined by IC. IC offers accurate phosphate quantification, which is crucial for quality assurance and regulatory compliance. While IC is usually capable of tolerating many interferences, complex sample matrices may contain additional ions that could impede phosphate identification. Proper handling and storage conditions are crucial because phosphorus compounds, particularly organophosphates, can deteriorate [28,141].

### 7.3. Gas Chromatography (GC)

GC is an analytical method for separating and analyzing substances that can evaporate without breaking down. It is frequently employed to determine and measure the constituents of a mixture [142]. Components of GC include a carrier gas and an inert gas, such as hydrogen, nitrogen, or helium, which is the mobile phase in GC, and it transports the sample down the column [142]. A tiny layer of liquid or polymer resting on an inert solid support within the column is the stationary phase. To improve separation efficiency, the column can be lengthy and coiled and is typically composed of glass or stainless steel [142]. It is through the injector that the sample is introduced into the GC system and evaporates into the carrier gas stream. Varied interactions between the chemicals in the sample and the stationary phase result in varied retention times. Following their passage through a detector as they leave the column, the separated compounds produce a signal according to the amount of each constituent. Mass spectrometers and flame ionization detectors (FID) are examples of common detectors [143].

Phosphorus compounds often have low volatility and are not thermally stable, making direct GC analysis challenging. The process of derivatization chemically modifies these compounds to improve their properties, allowing for more effective analysis [144]. A study by Farajzadeh et al. [145] investigated why certain organic compounds with carboxyl, hydroxyl, and amino functional groups are challenging for GC; it was determined that this was because of their physicochemical characteristics, which include low volatility, high polarity, and high water solubility [145]. The researchers found that proper sample preparation, which may involve concentration and derivatization techniques or an extraction clean-up step, is typically necessary to obtain the best analytical conditions. Some applications are in the analysis of pesticides, phosphorus, and pharmaceuticals [118,144]. To guarantee precise measurement of phosphorus-containing pesticides in biological and environmental samples, many of them undergo derivatization prior to GC analysis. Derivatization, in general, improves the volatility, stability, and detectability of phosphorus compounds, making them acceptable for GC analysis [118,145].

## 8. Electrochemical Methods

A collection of methods known as electrochemical analysis examines the connection between chemical reactions and electricity [146]. It is employed in the measurement of chemical species concentrations, the identification of reaction pathways, and the exploration of material attributes [146,147]. The capacity of electrochemical techniques to provide details regarding the oxidation and reduction (redox) characteristics of analytes is one of their main advantages. Applications include monitoring the environment and finding contaminants, heavy metals, and nutrients in the soil and water [148]. In the pharmaceutical industry, electrochemical methods are used to research medication interactions, developing new drugs, and maintaining quality [149,150]. Energy Storage examines the effectiveness and efficiency of supercapacitors, fuel cells, and batteries [49]. This review concentrates on the latest developments in flexible electrochemical biosensors that are integrated with a broad range of nanomaterials.

### 8.1. Potentiometry

The electrical potential, or voltage, of an electrochemical cell can be measured using potentiometer, an electrochemical analytical method that does not require a lot of current [151]. Potentiometry is very helpful for figuring out ion concentrations in different samples since the measured potential is closely correlated with the concentration of a particular ion in the solution [152]. The classifications include direct potentiometric analysis. This method uses the Nernst equation to measure the potential directly and connect it to the concentration of an ion in the solution [152,153]. The benefits of potentiometry include high selectivity, particularly when employing electrodes that are ion-selective [100]. Non-destructive throughout the measurement, the sample essentially stays unchanged [151]. Many measurements can be completed quickly and with little advance planning [154].

Phosphorus can be measured using potentiometry, especially when it comes to phosphate ions, which are the most prevalent and stable form of phosphorus in aqueous solutions [153,155]. Ion-selective electrodes (ISEs) that are sensitive to phosphate ions are commonly used in potentiometry analyses of phosphorus. To produce orthophosphate ions, the phosphorus-containing material is frequently digested to release all forms of phosphorus, including organic phosphorus and polyphosphates [155]. The phosphate concentration is determined by comparing the measured potential with the calibration curve [153,156]. To provide precise results, phosphate ISEs must be routinely maintained and calibrated. Potentiometry works well for measuring phosphate, although at very low concentrations, it could not be as sensitive as spectrophotometry or other techniques [152,157]. In environmental and agricultural applications where quick on-site analysis is advantageous, potentiometry offers a useful and accurate way to measure phosphorus in a variety of forms [157].

### 8.2. Voltammetry

**Figure 8:**
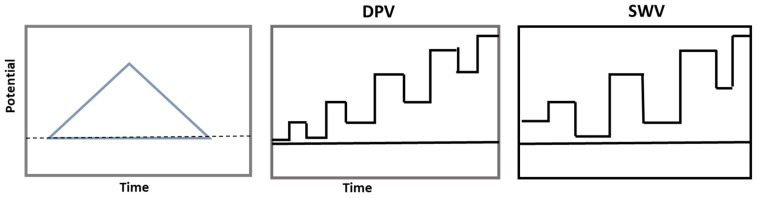
Diagrams representing the different types of voltammetry techniques.

Voltammetry is a dynamic electrochemical method that measures the current response in an electrochemical cell as a function of an applied potential (voltage) to examine redox (oxidation-reduction) reactions [158]. Voltammetry is a flexible and effective analytical chemistry technology that allows for the detection and quantification of a large variety of chemical species as well as deep insights into redox processes. Linear sweep voltammetry (LSV) is a popular voltammetry method, which involves sweeping the voltage of the working electrode linearly from an initial value to a final value [159]. Differential pulse voltage (DPV) overlays linear sweep potential with tiny potential pulses [160]. Current is measured just prior to and following overlays linear sweep potential with tiny potential pulses [161]. Voltammetry with Square Waves (SWV) provides the working electrode with a square wave potential modulation that alternates between two potential levels [162]. SWV is frequently employed in electrochemical sensing because it is quick and sensitive [163]. A diagram representing the different types of voltammetry techniques is shown in Figure 8.

Voltammetry applications include environmental monitoring identification of trace metals in soil, water, and air, such as lead, cadmium, and mercury [164,165]. The benefit of voltammetry is its high sensitivity; it can identify analyte concentrations as low as less than 10ppm [166]. To guarantee reliable results, meticulous control of the experimental parameters, such as the composition of the electrolyte and the cleanliness of the electrode surface, is necessary [167].

### 8.3. Conductometry

Conductometry is an analytical technique used to measure the electrical conductivity of a solution [68]. It is commonly employed in chemistry, particularly in the study of ionization reactions, titrations, and the determination of the purity of water or other solvents [168]. The electrical conductivity of a solution depends on the concentration of ions present in it; this is the principle of conductometry [68]. When an electrolyte dissolves in water, it dissociates into positive and negative ions, which can conduct electricity [169]. The dissociation constant is used to measure the conductivity at various concentrations to calculate the dissociation constants of weak acids or bases [68,169].

Phosphorus, especially as phosphate ions (PO_4_^3−^), can be analyzed with conductometry using indirect techniques that result in the creation of complex ions or precipitates [68,141]. The fundamental method is to change the phosphate ions into a form that modifies the solution’s conductivity such that it can be quantified. Phosphorus is frequently found as phosphate ions in soil or water samples. By adding a reagent that reacts with phosphate to generate an insoluble molecule, these ions can be precipitated. The concentration of phosphorus can be ascertained by measuring the difference in conductivity before and after the reaction [170]. A BaCl_2_ solution is used to titrate the phosphate ions in the sample. The addition of BaCl_2_ causes Ba^3+^ ions to combine with PO_4_^3−^ ions to generate Ba_3_(PO_4_)_2_, which lowers the concentration of free ions in the solution and, in turn, the solution’s conductivity drops as a result [141]. When the conductivity stops decreasing, meaning that all of the phosphate ions have precipitated out, the titration reaches its endpoint. The phosphorus concentration in the sample is correlated with the amount of reagent added until the endpoint [170,171]. In many different contexts, conductometric phosphorus analysis techniques are helpful, especially when a quick and accurate assessment of phosphate concentrations is required [172,173].

## 9. Nuclear Magnetic Resonance Spectroscopy

### Principles of NMR in Phosphorus Detection

**Figure 9:**
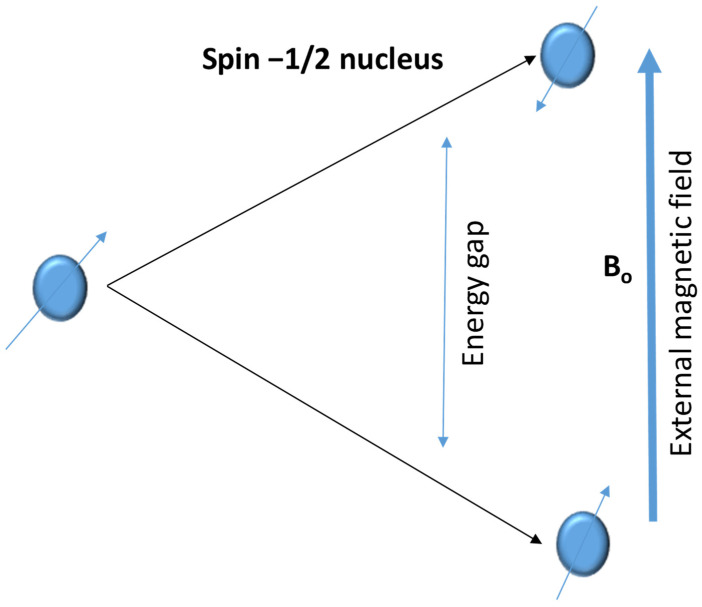
Diagram representing the different energies state in NMR analysis.

Phosphorus can be found and studied using NMR spectroscopy, especially when using ^31^P NMR spectroscopy [174,175]. With a nuclear spin of ½, phosphorus-31 (^31^P) is an NMR-active naturally occurring isotope of phosphorus. With special considerations for phosphorus, the principles of ^31^P NMR spectroscopy are comparable to those of other nuclei, such as ^1^H and ^13^C [174,175]. Fundamentals of ^31^P NMR for phosphorus is the identification of magnetic moment and nuclear spin; with a spin quantum number of ½, the ^31^P nucleus possesses a magnetic moment. As a result, the nucleus can align itself against or in line with an applied magnetic field. Depending on their surroundings, ^31^P nuclei can absorb RF radiation at a particular resonance frequency when exposed to a high magnetic field [176]. The electrical environment surrounding the phosphorus atom affects the chemical shift in ^31^P NMR [175]. The local electron density is influenced by variables including bonding, hybridization, and the electronegativity of nearby atoms, all of which change the resonance frequency [176]. Figure 9 shows a diagram representing the different energy states in NMR analysis.

Different phosphorus environments inside a molecule can be distinguished by NMR, which aids in the identification of functional groups and connections [177,178]. To obtain knowledge on pollution and nutrient cycles, it is also used to examine the amount of phosphorus present in environmental samples, such as soil and water [177]. ^31^P NMR is used in coordination chemistry to investigate the geometry of metal complexes, ligand exchange, and metal-phosphorus interactions [178]. Characterizing the coordination environment of phosphorus ligands in transition metal complexes is facilitated by this technique [179].

## 10. Emerging and Novel Techniques

Combining cutting-edge technologies and emerging methods for phosphorus assessment in environmental samples is transforming analytical capabilities [28]. Lab-on-a-chip devices are perfect for on-site applications and resource-constrained environments because they use microfluidic platforms to offer a quick, low-volume, and highly effective phosphorus analysis [100]. Nanosensors and nanoparticle-enhanced detection systems are two examples of advances brought about by nanotechnology-based techniques that greatly improve the sensitivity and selectivity of trace-level phosphorus detection [180]. Biosensors and bioreporters offer highly specific and eco-friendly phosphorus monitoring techniques by using biological recognition elements such as enzymes, bacteria, or DNA sequences [164]. When it comes to identifying bioavailable phosphorus fractions and providing information about its ecological effects, these biosensors are especially useful. When combined, these cutting-edge approaches offer strong, flexible ways to enhance phosphorus monitoring in intricate and changing environmental systems, potentially filling gaps in existing approaches and enabling proactive environmental management [4].

The involvement of nanotechnology in the determination of phosphorus in environmental samples is an established and growing field of environmental nanotechnology research [181]. It comes in the form of a supportive role of extraction of the phosphorus compounds prior to chromatographic analysis, or in the form of direct involvement in the sensing mechanism using various techniques [182]. Amiri et al. [182] demonstrated this when a coated stainless steel mesh (SSM) was employed for the adsorption of organophosphorus pesticides (OPP) in water and fruit juice samples [182]. The coating achieved by the sol-gel method comprised a mixture of oxidized carbon nanotubes (CNTs) and polyethylene glycol (PEG). The final product was a porous mesh with a rough surface and large surface area that enhances the sorption capacity of the material. The coated mesh were stacked in a syringe to form a solid phase extraction (SPE) cartridge, where, at optimum conditions, 70 mL of sample solution (diluted in deionized water) was passed for the adsorption of the OPPs, which were then eluted with only 0.8 mL of acetonitrile. This volume is much smaller than the volumes typically used in extraction techniques such as liquid-liquid extraction preparing for P analyses [183].

Sensing nano-based technologies have also been proposed. Talarico et al. [184] demonstrated a miniaturized sensor that employs carbon black nanoparticles (CBNPs) for the monitoring of phosphate in water bodies [184]. CBNPs are used to modify the surface of a screen-printed electrode. Then they eletrocatalytically enhance the reduction of the phosphomolybdate complex formed by the presence of phosphate and molybdate in solution to form electroactivity at the surface of the electrode. This electroactivity is then measured using a potentiostat to detect phosphate at low potentials, leading to a system that can be used for continuous monitoring of the environment [184]. The large surface area of CNTs was also exploited in a different study to immobilize cobalt oxide nanoparticles (CoONPs) for sensitive detection of phosphate in water [50,185]. The multi-walled CNTS (MWCNTs) were first modified with polybenzimidazole (PBI) prior to hosting CoONPs. Subsequently, a glassy carbon electrode (GCE) was coated with a suspension of CoONPs-PBI-MWCNTs and dried to form the working electrode for cycle voltametric measurement. This system takes advantage of the reported surface reduction-oxidation properties of transition metals oxides to directly react with phosphate anions in water [185]. This reaction forms a cobalt phosphate precipitate on the surface of the electrode, which presents a different electrochemical sensing mechanism to that reported by Talarico et al. [184]. The CoONPs-PBI-MWCNTs electrode was able to detect phosphate in water in the range of 0.1–100 nM, which is lower than the 0.1 µM eutrophication threshold, making the electrode suitable for phosphate monitoring [185].

## 11. Field-Deployable and On-Site Methods

### 11.1. Portable Analytical Instruments

On-site and field-deployable techniques for phosphorus determination have become more popular because they can offer quick and affordable monitoring in dynamic and distant settings [38]. The time and resources required for sample collection and laboratory analysis are decreased by the use of portable analytical tools, such as colorimeters and handheld spectrophotometers, which allow for rapid assessments of phosphorus levels in situ [7]. Frequently, these devices are complemented with reagent kits and simple protocols that make them accessible to non-specialist users. Sensor-based systems and microfluidic devices are examples of real-time monitoring technologies that provide continuous data acquisition and reveal temporal fluctuations in phosphorus concentrations [4]. These techniques are now even more reliable and efficient thanks to developments in optical and electrochemical sensors combined with wireless data transfer [186]. To solve the environmental issues related to phosphorus pollution, these field-deployable technologies are essential since they facilitate quick decision-making and flexible management techniques [38]. Benefits of portable analytical tools for phosphorus determination include quick on-site analysis, simplicity of use, and low sample preparation requirements [187]. They do have several drawbacks, though, such as a limited detection range, possible interference from other ions, and lesser sensitivity and accuracy when compared to laboratory-based methods [188]. Reliability of measurements can also be impacted by changes in environmental factors like temperature and turbidity. In order to guarantee consistent functioning, this equipment can also need regular calibration and maintenance. Although they are helpful for initial screening, more accurate laboratory techniques are frequently required for confirmatory analysis for research and regulatory purposes [189].

The molybdenum blue method is a mainstay, even in the construction of portable instruments for phosphorus determination in the environment. Researchers take advantage of the method’s established success to develop portable devices for environmental monitoring. The design of the instruments differs, with some taking the form of multi-layer microfluidic discs [189] and others a single microflow cell [78]. These systems rely on the assembly of individual parts such as infrared LED or laser module, a photodiode or photodetector, LCD screen, and a command interface to build a portable device [78].

The disc-based microfluidics system works by storing the dry reagents for the molybdenum blue reaction (except for phosphorus source) inside the discs. Once the phosphorus solution (sample) has been added, the disc system rotates to generate centrifugal forces that drive the solution to the reagents to form liquid blue complex in liquid form, which exhibits absorption properties at 880 nm (near infrared) [189]. The different disc layers perform different tasks, including ensuring air ventilation, providing the microchannels for fluid flow, storage of reagents, and sealing and prevention of leaks. The blue complex flows through the microchannel into the detection zone, which comprises a light emitting diode (LED) and a photodiode (PD) [78]. For incident light, the LED interacts with the blue complex in the detection zone of the disc and the transmitted light is measured by the PD to give results of the test on an LCD screen that is also part of the device. Centrifugation in this system eliminates any need to use pumps to propel externally loaded solutions. This contributes to the miniaturization and portability of the device. Phosphorus results obtained from this system showed very good agreement with those obtained from a UV-vis spectrophotomenter with just a 10 min reaction time. The LOD of this system was found to be 16 µg L^−1^ [189].

A cell-based phosphorus portable colorimeter has been reported, where the single cell was used as the reaction chamber for all the reagents needed for the formation of the phosphomolybdate complex [78]. This was made possible by installing a series of solenoid pumps, which were then filled with the solutions to feed into the cell to affect the molybdenum blue reaction. The fluid then passes between a laser module emitting light at 800 nm and photodetector to record the transmitted light. Signals generated during the testing could be plotted to display a real-time visualization of the process. This system was able to achieve LOD of 0.01 mg L^−1^ [189,190].

Existing commercial devices have also been investigated as portable analytical devices. The use of smartphones as potential portable devices for environmental monitoring due to the advancements made on their computing and sensing abilities has been reported [190]. Thus far, colorimetric sensors are touted as the most practical type of sensors for smartphone-based sensing applications [190]. However, Ai et al. [46] argue that there are quality control issues that are often not addressed in the smartphone-based P measurement methods, such as calibration curves that are constructed from a few data points and the lack of discussion on P concentrations around the limits set by regulatory guidelines among others [46]. Their own approach was to develop a method that used the smartphone camera and a machine learning (ML) algorithm to simplify the measurement process and eliminate the use of any other equipment that is meant to aid the measurement. This equipment includes LED light boxes [191], LED tool kits [192], UV light boxes [193], and infrared light boxes [188]. In their study, the group took a total of 1922 images of the molybdenum blue reaction under varied conditions, such as using cameras from different phones, different types of water samples, different ambient lighting conditions, and in different reaction containers. Red-green-blue values were extracted from the images and modelled using ML algorithms to estimate P concentrations, achieving a correlation coefficient of 0.97 for P concentrations between 0.01 and 1.0 mg L^−1^ and accuracies of 95.2% for P concentration less than 0.1 mg L^−1^ [46].

### 11.2. Real-Time Monitoring Technologies

In the field of analytical chemistry, paper-based analytical devices (PADs)—often in the form of microfluidic devices (µPAD)—are among the most common technologies for real-time monitoring of the environment as they offer qualities such as being energy unintensive, reduced chemical usage, and reduced sample pre-treatment requirements [39]. PADs can be underlined by different techniques for detecting analytes, such as electrochemistry and colorimetry, both of which have been widely reported for the detection of phosphorus in the environment [39,194].

For the colorimetric PADs for phosphorus detection, the proven method of molybdenum blue reaction is commonly exploited due to its simplicity and well-understood chemistry, providing sensitive and reliable detection of phosphorus [195]. This was illustrated in a study where Choudhary and Philip developed a paper-based colorimetric sensor for phosphate ion (PI, PO_4_^3−^) detection in wastewater samples [195]. The probe was prepared by dipping a strip of filter into an immobilizing agent and then adding brilliant green (BG) as a coloring agent followed by drying in an oven. Further soaking in a solution of ammonium molybdate was performed to load the complexing ion MoO^4−^. When the sensor was immersed in solutions containing PI, it changed color from yellow to green to indicate PI detection, with the intensity of the green color increasing with increasing PI concentration in the solution [195]. The yellow-green color change was detectable by the naked eye and confirmed by optical microscopy and UV-Vis spectroscopy (liquid probes without immobilization on paper), making the sensor an attractive alternative to expensive analytical equipment. FTIR characterization of the sensor before and after interacting with PI suggested the complexation of Mo and phosphorus atom via oxygen bridging, which led to the proposal of the mechanism where the Mo-P complex subsequently interacts with BG to affect the color change [194]. This was achieved within 5 min of immersing the sensor into the PI solution. The was able to achieve analytical merits such as LOD of 0.07 mg L^−1^, and selectivity towards PI in the presence of cations and anions at concentrations 10 times (13.6 mg L^−1^) that of PI in a multicomponent system [195].

**Figure 10:**
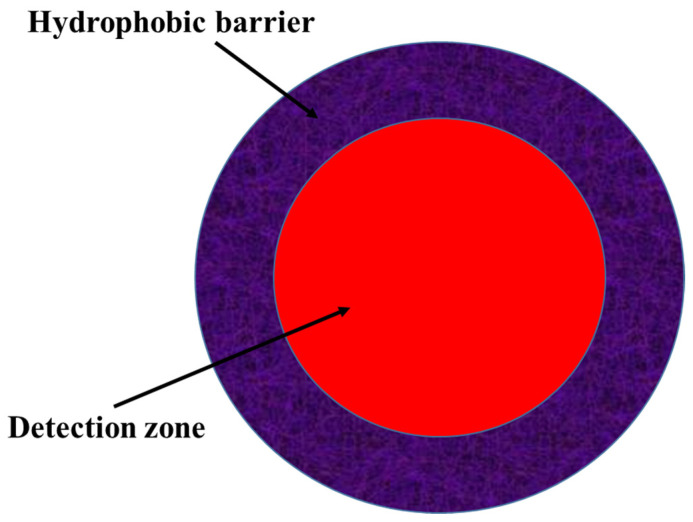
Image showing the hydrophobic barrier and detection zone of a µPAD.

Paper-based microfluidics (µPADs) refers to the science of producing devices from paper or another porous membrane that uses capillaries to handle and control small volumes of fluids (nL-µL) [196]. Several techniques exist for producing µPADs. Typically, µPADs function by having a hydrophobic barrier created around the detection zone to trap the analytical solution in the zone [194]. An example of this is shown in Figure 10, where a red solution is confined inside a blue hydrophobic barrier. For the quality of the analysis, it is important to have consistency in the thickness of the hydrophobic barrier and the detection zone; hence some of the µPADs are developed using more advanced techniques such as laser printers, inkjet printing, wax printing, lithography, etc., for precision and other advantages [196].

Electrochemistry-based PADs rely on the measurement of an electrochemical signal that is related to the detection of P in the sample. The role of a PAD in the sensor system, unlike in the colorimetric-based PADs, is not confined to reporting P. Rather, the PAD acts as a medium for extraction and pre-concentration of P from the sample. Zeitoun et al. [197] developed a PAD for P detection in soil samples by immobilizing an extractant (Mehlich-3) and ammonium molybdate onto a piece of filter paper [197]. Mehlich-3 is a well-known mixture of various reagents that can extract metal ions bound to P in soil and leave P free for uptake by other chemical agents [198]. In the study by Zeitoun et al. [197], Mehlich-3 was used in conjunction with ammonium molybdate tetrahydrate, which reacted with the free P to form a complex. In this way, the researchers managed to extract and concentrate P for cyclic voltametric determination using a commercial potentiostat after leaching the complex from PAD into a small amount of water [197]. Besides using a PAD for immobilization, molybdenum blue can be deposited directly onto the electrode via cyclic voltammetry for electrochemical determination of phosphorus in soil [198].

## 12. Quality Assurance and Quality Control (QA/QC)

The reliability of analytical techniques for phosphorus detection in environmental samples is largely dependent on QA/QC. For research to remain consistent and comparable, standardized procedures must be followed and approved reference materials must be used for calibration [104]. Method validation, which includes assessing sensitivity, accuracy, and precision to make sure the techniques yield accurate data over a range of sample matrices, is essential [171]. Precision measures the reproducibility of results under uniform conditions, whereas accuracy measures how closely measured values match the genuine value. The ability to identify traces of phosphorus in intricate environmental matrices is guaranteed by sensitivity, which is determined by the detection and quantification limits of the method [15]. For environmental monitoring and regulatory compliance, robust QA/QC practices are essential for reducing mistakes and boosting confidence in the data produced. These practices include regular instrument calibration, matrix-matching standards, and inter-laboratory comparisons [59].

The quality aspect of phosphorus detection in the environment is fundamental to decision-making about the state of soils and water bodies. Programmes such as inter-laboratory comparisons, using reference materials (RFs) and CRMs, and adherence to quality guidelines are some of the approaches that can be followed to achieve reliable data quality for phosphorus determination. CRMs can be used in the context of phosphorus determination to gauge the accuracy of the proposed method or they can be used for calibration purposes [182]. However, the use of CRMs in any way is not widely reported in research reporting on the determination of phosphorus in the environment, with only a few studies reporting their use during investigations [120]. Instead, for accuracy determinations, spike recoveries are widely performed, whereby the measured sample is spiked with a known concentration of the analyte and analyzed. Then the percentage concentration of the spiked sample relative to the expected concentration is calculated [122,182].

## 13. Comparative Analysis of Methods

Analytical methodologies differ greatly in their strengths and drawbacks [32,59]. When it comes to testing orthophosphate, traditional colorimetric techniques like the molybdenum blue method are commonly employed because of their ease of use, affordability, and dependability [76]. Nevertheless, their capacity to quantify total phosphorus, which encompasses both organic and particulate forms, is restricted, and they can be subject to interference from other compounds [36,61]. Measurements of total phosphorus can be made with great sensitivity and precision using ICP-OES and ICP-MS [14,199]. These methods are effective for thorough analysis, but they need specialized knowledge, costly equipment, and extensive sample preparation [4]. Table 6 compares the costs and suitability of many analytical methods, including sample matrices, for phosphorus determination. Another reliable technique that works well for differentiating and quantifying distinct phosphorus species is ion chromatography [199]. However, it also takes a lot of time and requires complex equipment. However, sophisticated spectroscopic methods such as Raman Spectroscopy and LIBS require calibration and offer quick, in situ analysis with little sample preparation [12].

Case studies that contrast approaches in actual applications show how selecting one methodology over another can have practical effects [76]. For instance, a study on agricultural runoff may contrast the accuracy and efficiency of colorimetric and ICP-OES techniques [10,76]. In field settings, the colorimetric approach may be preferred due to its ease of use and affordability, as it offers sufficient information for controlling fertilizer application and averting eutrophication [32]. On the other hand, for in-depth analysis and research reasons, ICP-OES would be chosen in a laboratory setting due to its higher complexity and cost, as it provides a more thorough understanding of all phosphorous forms present [35].

Phosphorus’s speciation may be examined in detail using sequential extraction and ICP-MS, which can shed light on the mineral’s bioavailability and its effects on the environment [15]. Although comprehensive, this method requires a lot of resources. In contrast, a more pragmatic evaluation that strikes a balance between efficiency and detail can make use of a mix of colorimetric techniques and ion chromatography [6]. Comparing biosensor performance with conventional chemical approaches in wastewater treatment context could highlight improvements in real-time monitoring capabilities [58]. Biosensors have the potential to facilitate more dynamic treatment process management due to their high specificity and quick response times, while older approaches may provide more reliable and confirmed results but have slower data availability [6]. Researchers and practitioners can choose the best approach for their particular environmental phosphorus analysis needs by being aware of the advantages and disadvantages of each technique [11].

## 14. Regulatory Standards and Guidelines

Because phosphorus contributes to water eutrophication and the ensuing deterioration of ecosystems, regulations and recommendations for phosphorus assessment in environmental samples have become more stringent [1]. To reduce their negative effects on the environment, a number of international organizations, including the European Union (EU) and the United States Environmental Protection Agency (USEPA), have set acceptable phosphorus levels for surface and wastewater [200]. These laws require that phosphorus levels be monitored using extremely sensitive and accurate analytical methods. The demand for accurate, low-level phosphorus quantification in compliance with regulations has been met by recent improvements in detection limits and accuracy in techniques including ICP-OES, ICP-MS, XRF, and spectrophotometric approaches [10]. Continuous improvements in analytical techniques are essential for complying with changing environmental requirements and promoting sustainable water management practices, as the necessity for accurate phosphorus measurement grows [9,201].

Adhering to several standards can be expensive and complex, especially for small and medium-sized businesses (SMEs) [200,202]. Organizations must remain aware and flexible since regulatory standards are modified frequently to take into account new technology, scientific advancements, and public expectations [202]. For industries to function in a safe, moral, and sustainable manner, regulations and standards are essential. In addition to safeguarding the environment, public health, and safety, they offer a framework for innovation [200,202]. Phosphorus analysis is essential in many sectors, such as water quality management, food and beverage production, agriculture, pharmaceuticals, and environmental monitoring [5,35]. Both national and international standards have been developed to guarantee the precision, dependability, and comparability of phosphorus analysis across various laboratories and applications [203]. These guidelines address how to measure the amount of phosphorus present in several matrices, including food, water, soil, and fertilizers [14,204].

## 15. Future Directions and Research Trends

Technological developments and interdisciplinary techniques are expected to have a major impact on future directions and research trends in the analysis of phosphorus in environmental samples [14,205]. New technologies have the potential to improve phosphorus analysis’s accuracy, effectiveness, and application while resolving existing issues and creating new opportunities [9]. Future technological developments include the fusion of biosensors, nanotechnology, and sophisticated spectroscopic methods [2,25,205]. With the use of nanotechnology, it may be possible to create extremely selective and sensitive nanosensors that can find traces of phosphorous in intricate matrices [205]. Real-time on-site monitoring could be made possible by integrating these nanosensors with portable devices [206]. These biosensors can provide more precise information regarding phosphorus speciation in environmental samples due to their high selectivity for various forms of phosphorus [75]. Furthermore, improvements in spectroscopic techniques, such Raman spectroscopy and LIBS, should improve the capacity to analyze phosphorus in situ and with little sample preparation [6]. These techniques may offer quick, non-destructive phosphorus analysis of soils, sediments, and aquatic environments [53].

Real-time on-site monitoring could be greatly improved by developments in portable, miniature analytical instruments, such as lab-on-a-chip technologies and handheld spectrometers [207]. Furthermore, incorporating machine learning and artificial intelligence into data processing could increase precision, lower errors, and enable automated analysis [191]. Sustainable phosphorus monitoring would be aided by the development of green analytical chemistry techniques, such as reagent-free or environmentally friendly procedures. Furthermore, more thorough environmental studies may be possible with enhanced multi-element detection capabilities in methods like ICP-MS and LIBS [122]. To guarantee data comparability and dependability, standardization and harmonization of procedures across various regulatory frameworks should also be given top priority. Lastly, methods that are both affordable and scalable need to be investigated to facilitate extensive monitoring initiatives, especially in areas with scarce resources [197].

To advance phosphorus analysis, interdisciplinary approaches and collaborations are becoming increasingly important [4]. The creation of novel techniques that make use of the advantages of each discipline can result from the cooperation of chemists, biologists, environmental scientists, and engineers [36]. For example, integrating biological assays with chemical extraction procedures can enhance the identification and measurement of different types of phosphorus [27]. To more precisely forecast the behavior and effects of phosphorus, environmental modelling and data science can also be extremely important [205]. They do this by combining analytical data with environmental characteristics. Additionally, collaborations with agricultural scientists can assist in customizing analytical techniques to address particular issues with crop yield and soil fertility, resulting in more sustainable agriculture practices. Moreover, partnerships between universities, businesses, and government organizations can help translate scientific discoveries into useful applications. These collaborations can guarantee that new technologies meet regulatory requirements, are commercially feasible, and are supported by science. To address the global difficulties associated with phosphorus management, such as reducing eutrophication, maximizing fertilizer use, and enhancing water quality, an interdisciplinary and cooperative approach is necessary.

## 16. Conclusions

Significant improvements that have improved the precision, sensitivity, and effectiveness of phosphorus detection are highlighted in the evaluation of recent innovations in analytical techniques for phosphorus determination in environmental samples. Although phosphorus is an essential component of agricultural activities and natural ecosystems, too much of it in water bodies can cause eutrophication and other environmental problems. Phosphorus levels in diverse environmental matrices must therefore be monitored and managed using accurate and trustworthy analytical techniques. The focus of recent developments has been on enhancing conventional techniques like spectrophotometry and colorimetry to make them less susceptible to interference and more sensitive. Faster analysis times and lower detection limits are now possible thanks to advancements in reagent formulations and detection technologies. Furthermore, in situ measurements have been made easier by the introduction of portable and automated instruments, which allow for real-time monitoring of phosphorus levels in field settings. Modern methods with great precision, resilience, and the capacity to detect many elements at once, like ICP-MS, have become more and more popular. These techniques are especially useful for complicated environmental samples that need in-depth examination. Furthermore, these methods’ usefulness and accuracy in detecting phosphorus traces have been further enhanced by their integration with pre-concentration and separation processes. In the field of phosphorus analysis, emerging technologies such as biosensors and sensors based on nanomaterials offer great potential. High sensitivity, selectivity, miniaturization, and real-time monitoring are all possible with these techniques. The capacity to monitor and control phosphorus levels in the environment has been greatly improved by the latest advancements in analytical techniques for phosphorus determination.

## Figures and Tables

**Table 1:** Environmental impact and significance of phosphorus in the environment.

Environmental Matrix	Source of Phosphorus	Transport Mechanism	Environmental Impact	Significance and Regulatory Concern	References
Water	Agricultural runoff, wastewater discharge, industrial effluents, atmospheric deposition	Surface runoff, leaching, groundwater flow	Eutrophication, algal blooms, hypoxia, biodiversity loss	Leads to oxygen depletion, fish kills, and water quality degradation; regulatory limits set for total phosphorus in surface waters	[37]
Soil	Fertilizer application, manure, mining activities, weathering of phosphate rocks	Adsorption-desorption, leaching, runoff	Soil degradation, phosphorus accumulation, loss of soil fertility	Excess P leads to nutrient imbalance, affecting crop growth and microbial communities	[38]
Air	Atmospheric deposition from industrial emissions, biomass burning, wind erosion of soil particles	Dry and wet deposition, particulate matter transport	Phosphorus-containing aerosols affecting cloud formation, air quality, and nutrient deposition in remote ecosystems	Limited research on P cycling in the atmosphere; potential implications for climate change and global biogeochemical cycles	[39]
Human Health	Contaminated drinking water, food chain bioaccumulation, occupational exposure	Ingestion, inhalation, dermal contact	Metabolic disorders, kidney disease, cardiovascular issues, potential carcinogenic effects	Regulatory bodies monitor P levels in drinking water; high intake linked to adverse health effects, requiring dietary and water quality controls	[40]
Ecosystems and Biodiversity	Disruptions in nutrient cycling, invasive species proliferation	Biotic uptake, trophic transfer	Loss of aquatic and terrestrial biodiversity, disruption of ecological balance	Requires integrated nutrient management and sustainable agricultural practices	[32]

**Table 2:** Sample collection and preservation across different water matrices.

Water Matrix	Sampling Method	Container Type	Preservation Method	Holding Time	References
Surface Water (Rivers, Lakes, Reservoirs)	Grab sampling at mid-depth using a clean polyethylene or glass bottle	High-density polyethylene (HDPE) or glass bottle	Store at 4 °C in the dark; acidify to pH < 2 with H_2_SO_4_ for phosphorus analysis	28 days (filtered samples), 48 h (unfiltered)	[46]
Groundwater	Pump sampling, avoiding stagnant water	Borosilicate glass or HDPE bottle	Keep samples at 4 °C; acidify if required	28 days (filtered), 48 h (unfiltered)	[47]
Wastewater (Industrial, Municipal Effluent)	Composite sampling using an automatic sampler or manual collection	HDPE or glass bottle	Store at 4 °C; acidify to pH < 2 for dissolved phosphorus	28 days	[34]
Rainwater	Direct collection in clean glass or HDPE bottles	Glass or HDPE bottle	Refrigerate at 4 °C; analyze as soon as possible	24 h	[48]
Seawater	Grab sampling, ensuring minimal contamination	Polycarbonate, HDPE, or glass bottle	Store at 4 °C; acidify with HCl to prevent microbial activity	28 days	[49]
Acid Mine Drainage (AMD)	Grab sampling using an acid-resistant container	HDPE or Teflon bottle	Store at 4 °C; acidify with HNO_3_ or H_2_SO_4_ to pH < 2	14–28 days	[50]

**Table 3:** Extraction techniques for phosphorus analysis in environmental samples.

Extraction Technique	Description	Advantages	Limitations	Applicable Matrix	References
	Chemical Extraction Methods				
Olsen Method	Uses sodium bicarbonate (NaHCO_3_) for alkaline soils	Simple, widely used, suitable for alkaline soils	Not suitable for acidic soils	Alkaline soils	[59]
Bray and Kurtz P1 Method	Uses ammonium fluoride (NH_4_F) and hydrochloric acid (HCl) for acidic soils	Effective for acidic soils, relatively quick	Not suitable for alkaline soils, potential reagent hazards	Acidic soils	[60]
Mehlich-3 Extraction	Uses a mixture of acids and chelating agents	Universal method, suitable for a wide range of soils	Requires careful handling and calibration	Various soil types	[61]
Morgan Extraction	Uses sodium acetate buffered at pH 4.8	Suitable for neutral to slightly acidic soils	Limited applicability to other soil types	Neutral to slightly acidic soils	[36,62]
	Physical Extraction Methods				
Sequential Extraction	Series of extractions to fractionate phosphorus into different forms	Detailed information on phosphorus forms	Labor-intensive, time-consuming	Soils, sediments, wastewater sludges	[37,63]
Soil Washing	Physical agitation with water or a chemical solution	Effective for remediation, removes excess phosphorus	Can alter soil structure, may require large volumes of water	Contaminated soils	[64]
Ultrasonication	Uses ultrasonic waves to enhance extraction	Improves efficiency of chemical methods, breaks down aggregates	Requires specialized equipment	Soils, sediments	[65]
	Advanced Extraction Techniques				
MAE	Uses microwave energy to heat the extraction solvent and sample	Faster extraction, reduced solvent usage	Requires specialized microwave equipment, potential safety concerns	Soils, sediments, sludges	[53]
SFE	Uses supercritical CO_2_, often with co-solvents	Environmentally friendly, high extraction efficiency	High initial cost, requires expertise	Soils, sediments	[58]
ASE	Uses high temperature and pressure to enhance extraction	Reduces time and solvent consumption, highly automated	High initial cost, complex operation	Soils, sediments, plant materials	[66]
Electrochemical Methods	Uses electric fields to enhance phosphorus mobility and extraction	Can improve recovery rates, potentially less chemical usage	Requires specialized equipment, not widely used	Contaminated soils, sediments	[67]
Nanotechnology-Based Methods	Uses nanoparticles to selectively bind and extract phosphorus	High specificity and sensitivity, potential for miniaturization	High cost, potential environmental impact of nanoparticles	Complex environmental matrices	[68]

**Table 4:** Colorimetric methods and digestion methods for phosphorus analysis.

Method	Application	Advantages	Limitations	Volume of Acids Used	Time	Reference
Molybdenum Blue Method	Water, soil, and sediment samples	High sensitivity, reliable, well-established	Interference from other ions, reagent handling	Low to moderate, typically <10 mL	1–2 h	[9,38]
Ascorbic Acid Method	Water, soil extracts	Simple, reliable, cost-effective	Sensitive to sample matrix, potential reagent instability	Low, typically <10 mL	1–2 h	[54]
Vanadomolybdophosphoric Acid Method	Soil and plant tissue samples	Simple, rapid analysis	Less sensitive than Molybdenum Blue, potential interference	Moderate, typically <20 mL	1–2 h	[75]
Persulfate Digestion	Total phosphorus in water, soil, and sediment samples	Converts all forms of phosphorus to orthophosphate	Requires heating and pressure, safety concerns	High, typically 10–50 mL	1–3 h (including digestion)	[76]
Aqua Regia Digestion	Soil and sediment samples	Effective for complex matrices, releases bound phosphorus	Harsh conditions, potential for loss of volatile phosphorus	High, typically 20–50 mL	2–4 h (including digestion)	[10]
Sequential Extraction	Fractionation of phosphorus in soil and sediments	Detailed information on phosphorus forms, high specificity	Time-consuming, multiple steps, potential for cross-contamination	Variable, depending on steps, typically 10–50 mL per step	4–24 h (multiple steps)	[63]

**Table 5:** Comparisons of total phosphorus concentrations reported from different places around the world.

Location	Extraction Method	Type of Sample	Percentage Recovery	Extraction Time	Method of Measurement	Total Phosphorus Concentration	References
University of Arkansas, Fayetteville, AR	Mehlich III (M3)	Soil	_	5 min	ICP-OES, P-NMR and MS	1–675 mg/kg	[61]
Yuanyang County, Henan Province, China	Olsen-P, Sequential extraction	Soil	_	30 min	Calorimetry blue method	12–358 mg/kg	[81]
Berlin, Germany	Microwave digestion, reflux digestion	Sewage sludge	80–121%	30–60 min	ICP-OES, ICP-MS, XRF	0.1–2.2 g/kg	[10]
Thessaloniki, Greece	Olsen and Mehlich-3 Method	Soil	_	5–30 min	ICP-OES and calorimetry	_	[71]
China	Acid digestion	Water	99.20–100.1%	30–50 min	Calorimetry (spectrophotometer)	31.1–55.5 μg/g	[9]
Guangxi University, China	Microwave digestion	Soil and Sludge	95.7–111.2%	10 min	headspace gas chromatography (HS-GC) and Colorimetric method	85.9–1584 mg/kg	[8]
Manitoba, Canada	Olsen, Mehlich 3, CaCl_2_, and water extraction methods	Soil	_	5–30 min	ICP-OES, Calorimetry	1.0–125.8 mg/kg	[38]
Zimbabwe	Mehlich-3 extractant	Soil	91–108%	5 min	Calorimetry, ICP-OES	7.94–31 mg/kg	[82]
Kielce, Poland	Sequential extraction	Plants	_	16 h	Calorimetry	_	[37]
Toyo, Asakura, Japan	Ultrasonic washing machine method	River water	_	5 min	Calorimetry (molybdenum blue method)	0.068–0.541 mg/L	[65]
Umaria district of Madhya Pradesh, India	XRF and FTIR preparation method	coal and coal ash.	_	20–60 min	Fourier Transform Infrared spectroscopy (FTIR), (XRF)	135.3–569.9 ppm	[17]

**Table 6:** Comparisons of the costs and applicability of various analytical techniques for phosphorus determination.

Technique	Capital Cost	Operational Cost	Sensitivity	Sample Matrix	Advantages	Disadvantages	References
ICP-OES	High	Moderate	High	Water, Soil, Sediments	Multi-element analysis, high throughput	High initial cost, requires skilled personnel	[105]
ICP-MS	Very High	High	Very High	Water, Soil, Sediments, Biological Samples	Extremely sensitive, low detection limits	Expensive, matrix interferences, complex sample preparation	[120]
UV-Vis Spectroscopy	Low	Low	Moderate	Water, Wastewater	Simple, cost-effective, rapid analysis	Lower sensitivity, potential interferences	[84]
Raman Spectroscopy	Moderate	Low	Moderate	Solid Minerals, Powders, Thin Films	Non-destructive, minimal sample prep	Fluorescence interference, limited sensitivity in some matrices	[83]
LIBS	Moderate	Moderate	Moderate-High	Solid Minerals, Sludges, Soil	Rapid analysis, minimal sample prep	Calibration required, matrix effects	[106]

## Data Availability

Not applicable.
